# Impact of a Four-Week NCAA-Compliant Pre-Season Strength and Conditioning Program on Body Composition in NCAA Division II Women’s Basketball

**DOI:** 10.3390/jfmk10030266

**Published:** 2025-07-15

**Authors:** Zacharias Papadakis

**Affiliations:** Human Performance Laboratory, Department of Health Sciences and Clinical Practice, College of Health Professions and Medical Sciences, Barry University, Miami Shores, FL 33161, USA; zpapadakis@barry.edu; Tel.: +1-305-899-3573

**Keywords:** high-intensity training, body fat, linear mixed models, NCAA regulations, bioelectrical impedance, female athletes, short-duration intervention

## Abstract

Background: Pre-season training is pivotal for optimizing athletic performance in collegiate basketball, yet the effectiveness of such programs in improving body composition (BC) under NCAA-mandated hourly restrictions remains underexplored. The aim of this study was to evaluate the impact of a four-week, NCAA Division II-compliant strength and conditioning (SC) program on BC in women’s basketball. Methods: Sixteen student athletes (20.6 ± 1.8 y; 173.9 ± 6.5 cm; 76.2 ± 20.2 kg) completed an eight-hour-per-week micro-cycle incorporating functional conditioning, Olympic-lift-centric resistance, and on-court skill development. Lean body mass (LBM) and body-fat percentage (BF%) were assessed using multi-frequency bioelectrical impedance on Day 1 and Day 28. Linear mixed-effects models were used to evaluate the fixed effect of Time (Pre, Post), including random intercepts for each athlete and covariate adjustment for age and height (α = 0.05). Results The LBM significantly increased by 1.49 kg (β = +1.49 ± 0.23 kg, t = 6.52, *p* < 0.001; 95% CI [1.02, 1.96]; R^2^ semi-partial = 0.55), while BF% decreased by 1.27 percentage points (β = −1.27 ± 0.58%, t = −2.20, *p* = 0.044; 95% CI [−2.45, −0.08]; R^2^ = 0.24). Height positively predicted LBM (β = +1.02 kg/cm, *p* < 0.001), whereas age showed no association (*p* > 0.64). Conclusions: A time-constrained, NCAA-compliant SC program meaningfully enhances lean mass and moderately reduces adiposity in collegiate women’s basketball athletes. These findings advocate for structured, high-intensity, mixed-modality training to maximize physiological readiness within existing regulatory frameworks. Future research should validate these results in larger cohorts and integrate performance metrics to further elucidate functional outcomes.

## 1. Introduction

In terms of strength and conditioning (SC), the pre-season is a pivotal phase for basketball athletes, bridging off-season and in-season competitive periods [1,2,3,4,5]. For NCAA Division II women’s basketball, this phase mandates meticulous programming to enhance physical and psychological preparedness within stringent NCAA regulatory constraints (e.g., max 4 h/day, 20 h/week) [6,7,8,9,10,11,12,13,14,15]. While SC protocols rightly prioritize anaerobic, power, and aerobic capacities [1,9,16,17], limited NCAA training hours necessitate maximally effective methods targeting crucial physiological variables [8,14,15,18]. Despite this, such programs’ transient influence on body composition remains inadequately quantified.

Body composition (BC), particularly lean body mass (LBM) and body fat percentage (BF%), critically determines athletic performance [18,19]. These parameters influence aerobic/anaerobic power, physical capacity, power-to-weight ratio, agility, and injury resilience [1,9,17,18,19,20,21,22,23]. However, pre-season and in-season BC changes in collegiate women’s basketball are often minimal, frequently showing no significant impact on performance or playing time [1]. Most studies report minor LBM and BF% shifts, with limited evidence directly linking them to on-court performance. Investigations into women’s basketball typically show slight, often insignificant, BF% decreases from pre- to in-season/post-season (average changes < 2), suggesting inconclusive pre-season training effects on BC [1,20,24,25,26,27,28].

Empirical evidence on pre-season BC changes varies. Ladwig et al. (2013) observed a 1.8 BF% reduction and 0.9 BM decrease in a Division I women’s team, implying LBM gain. Other research reports greater BF% reductions (up to 3.49) [29], alongside BM increases (1–1.5 kg) due to LBM gains [30]. A recent non-NCAA study in professional Greek National A2 Female Division documented a 1.18 BF% decrease pre- and post-pre-season [17]. Similarly, comparisons of three pre-season training programs (heavy, moderate, light) showed BF% decreases of 0.3, 1.29, and 0.39, respectively [16]. At the NBA level, in-season phases showed modest LBM increases and fat mass decreases compared to pre-season [22].

Diverse BC tracking methods (e.g., DEXA, BIA, skinfolds) used by SC coaches yield varying absolute results but similar trends of minimal change across pre-season to in-season transitions [18,22,31]. Bioelectrical impedance analysis (BIA) is widely used in SC due to its convenience and non-invasiveness for estimating LBM and BF%. However, BIA tends to underestimate BF% and overestimate LBM compared to dual energy X-ray absorptiometry (DEXA) [31,32,33].

Prior research emphasizes SC training’s importance in eliciting physiological adaptations, particularly augmenting LBM and reducing BF% [2,3,9,34,35], with systematic reviews confirming resistance training efficacy for lean mass accrual in elite female athletes even with short-term interventions [1,36,37]. Despite this, empirical evaluations of short-duration programs specifically within NCAA regulations remain scarce, as highlighted by systematic reviews identifying limited evidence for structured SC interventions in women’s team sports under constrained timelines [1,36,37].

Physiologically, the anticipated improvements in body composition are underpinned by distinct mechanisms. The accretion of LBM is primarily driven by a net positive muscle protein balance, where the rate of muscle protein synthesis exceeds muscle protein breakdown. High-intensity resistance training, particularly involving multi-joint, large muscle group exercises like those in an Olympic-lift-centric program, serves as a potent stimulus for muscle protein synthesis [36,38,39,40,41]. In female athletes, this response is modulated by a complex interplay of hormones, including growth hormones and insulin-like growth factor 1 (IGF-1), which are acutely elevated post-exercise and facilitate tissue repair and growth [36,42,43,44]. Concurrently, the reduction in BF% is a consequence of increased energy expenditure and enhanced fat oxidation. The combination of intense resistance exercise and metabolic conditioning creates a significant caloric deficit and elevates post-exercise oxygen consumption, leading to sustained energy use after the training session concludes [45,46,47]. Furthermore, chronic adaptation to such training can improve mitochondrial density and efficiency, enhancing the body’s capacity to utilize fatty acids as a fuel source, both at rest and during exercise [48,49,50].

These integrated physiological responses provide the theoretical basis for expecting favorable body composition shifts even within a constrained timeframe. Few studies have isolated short-term, NCAA-regulated pre-season intervention effects, leaving coaches without robust, evidence-based strategies to maximize physiological adaptations within limited timeframes [1,6,8,13,15,51,52,53]. The present study addresses this imperative by pragmatically evaluating a four-week pre-season SC program’s efficacy in improving LBM and BF% in NCAA Division II women’s basketball. Therefore, the primary and explicit hypothesis of this investigation was that a four-week, NCAA-compliant pre-season SC program would induce a statistically significant increase in LBM and a concurrent, statistically significant decrease in BF% in NCAA Division II female basketball players.

## 2. Materials and Methods

### 2.1. Participants

Sixteen NCAA Division II female basketball players (age: 20.56 ± 1.79 years; height: 173.88 ± 6.46 cm; body mass: 76.19 ± 20.16 kg) participated in this observational study. Recruitment aligned with SC coaches’ annual periodization plan. Due to stringent NCAA Division II regulations [15], a convenience sample was utilized. All available student athletes who were physically fit per NCAA regulations and willing to participate were included. Exclusion criteria comprised recent musculoskeletal injuries or non-compliance with established training protocols. Institutional review board approval and informed consent were obtained (Date:1/30/2022, No: 1851725-2).

### 2.2. Four-Weeks Pre-Season Training Protocol

The four-week pre-season training program, commencing 2 September (one week post-Fall semester start), strictly adhered to NCAA Division II regulations [15]. It was capped at 8 h/week: 4 h of on-court skill work and 4 h of SC. Consistent with SC coaches’ periodization, this regimen aimed to enhance strength, endurance, and basketball-specific skills for in-season performance.

Conditioning (06:45–07:15 h, Mon, Tue, Thu, Fri) involved functional core movements (e.g., sandbag carries, bear crawls) and the “Running Game” (12–16 lap timed circuit). Immediately following (07:15–08:00 h, same days), strength sessions implemented a push–pull–leg sequence, integrating Olympic-style lifts (e.g., cleans, jerks, overhead press) and concluding with a comprehensive core series (e.g., Russian twists, medicine-ball slams, sit-ups).

Basketball-specific skill training occurred three afternoons/week (16:00–17:00 h, Mon, Wed, Thu). Practices emphasized full-court drills, shooting progressions, five-on-five play, and defensive schematics, focusing on offensive execution, shot accuracy, overall conditioning, and tactical defense. Collectively, this meticulously periodized regimen progressively improved physical capacity and sport-specific proficiency, preparing players for competitive season demands.

### 2.3. Body Composition Assessment

Body composition metrics were obtained via multi-frequency BIA (TANITA MC-780U, Tokyo, Japan). Assessments were performed immediately pre- and post-intervention, aligning with SC coaches’ assessment schedule. The device provided estimates for LBM and BF%. All measurements adhered to rigorously standardized conditions per the TANITA operating manual, including avoiding strenuous exercise, excessive food/fluid intake, or dehydration, and replicating conditions across assessments. Data collection occurred on the team’s first and last pre-season days. This device has demonstrated high test–retest reliability for body composition metrics in athletic populations (Intraclass Correlation Coefficient > 0.98), making it a suitable instrument for tracking longitudinal changes in applied settings [54,55,56,57].

### 2.4. Statistical Analysis

Effects on LBM and BF% from the short-term, NCAA-regulated pre-season SC program were evaluated using a convenience sample of *n* = 16 participants (32 total observations across pre- and post-intervention time points). This sample size was dictated by NCAA regulations concerning pre-season activities precluding a priori sample size determination. Notwithstanding this constraint, a sensitivity power analysis (G*Power version 3.1.9.6) determined the statistical power of the available sample. For linear mixed model (LMM) analysis (*α* = 0.05) with two fixed-effect predictors, achieved power (*β* ≈ 0.80) indicated detection of a large effect (*f* = 0.80) [58].

Linear mixed models (LMMs) were employed (GAMLj module, Jamovi version 2.6.44) [59] to account for individual differences and potential covariates. LMMs were chosen for their robustness with repeated measures, accommodating observation dependency and modeling individual variability via random effects [60,61,62]. Each model included Time (Pre vs. Post) as a fixed effect to estimate mean change. Participant ID served as a clustering variable with a random intercept to account for non-independence of repeated observations and individual baseline variability. Age (years) and Height (cm) were included as centered, continuous fixed covariates to control for confounding and aid intercept interpretation [63,64,65].

Two distinct LMMs were developed: one for LBM and another for BF%. Models used a linear link function and restricted maximum likelihood (REML) estimation, optimizing parameter estimates for smaller samples. Satterthwaite approximation estimated denominator degrees of freedom for fixed effects tests. A compound symmetry covariance structure was assumed for random effects, providing a parsimonious approach for limited repeated measurements. To ensure the validity of the models’ inferences, fundamental statistical assumptions were rigorously examined. The LMM framework requires that the model’s residuals are normally and independently distributed and exhibit homogeneity of variance (homoscedasticity) across the range of predicted values. While the random intercept structure inherently addresses the assumption of independence for observations nested within participants, normality and homoscedasticity required explicit verification. Consequently, these assumptions were evaluated using a combination of statistical tests (e.g., Shapiro–Wilk) and visual inspection of diagnostic plots, including Q-Q plots of the residuals and scatterplots of residuals versus predicted values. Model fit was assessed via Akaike’s Information Criterion (AIC) and Bayesian Information Criterion (BIC), with lower values indicating superior fit. Post hoc pairwise comparisons (Pre vs. Post) for LBM and BF% used Bonferroni adjustment to control Type I error. Effect sizes (Cohen’s *d*) were calculated as:

$$d=\frac{t}{\sqrt{N}}$$

Cohen’s *d* was interpreted as small (0.20), medium (0.50), or large (0.80) [58]. Marginal and conditional *R*^2^ values quantified variance explained by fixed effects alone and the full model, respectively [66,67]. Fundamental model assumptions (normality of residuals via Shapiro–Wilk tests/Q-Q plots; homogeneity of variance via residual plots) were rigorously examined. All analyses were performed at *α* = 0.05 [68,69,70], with graphical representations from Jasp (version 0.19.3).

## 3. Results

### 3.1. Descriptive Statistics

The cohort comprised 16 NCAA Division II female basketball athletes. Full descriptive statistics, including standard deviations and confidence intervals, are detailed in Table 1.

### 3.2. Inferential Statistics

#### 3.2.1. Lean Body Mass (LBM)

The LMM for LBM converged successfully using the bobyqa optimizer. The random-intercept structure significantly improved model fit compared to an ordinary least-squares alternative (ΔAIC = 32.31; LRT = 47.29, *df* = 1, *p* < 0.001). Analysis revealed substantial variability in baseline LBM among participants, as indicated by the random intercept variance for Participant ID (Variance = 19.72, SD = 4.44). The intraclass correlation coefficient (ICC = 0.98) further suggested that a considerable proportion of the total variance in LBM was attributable to inter-individual differences. The overall model, incorporating both fixed and random effects, explained a very high proportion of the variance in LBM (Conditional R^2^ = 0.99), while the fixed effects alone accounted for a substantial amount (Marginal R^2^ = 0.68). Diagnostic checks of the residuals confirmed the model’s assumptions: residuals were normally distributed (Kolmogorov–Smirnov *D* = 0.10, *p* = 0.840; Shapiro–Wilk *W* = 0.95, *p* = 0.105), and visual inspection of residual plots indicated homoscedasticity with no apparent deviations from normality.

**Figure 1:**
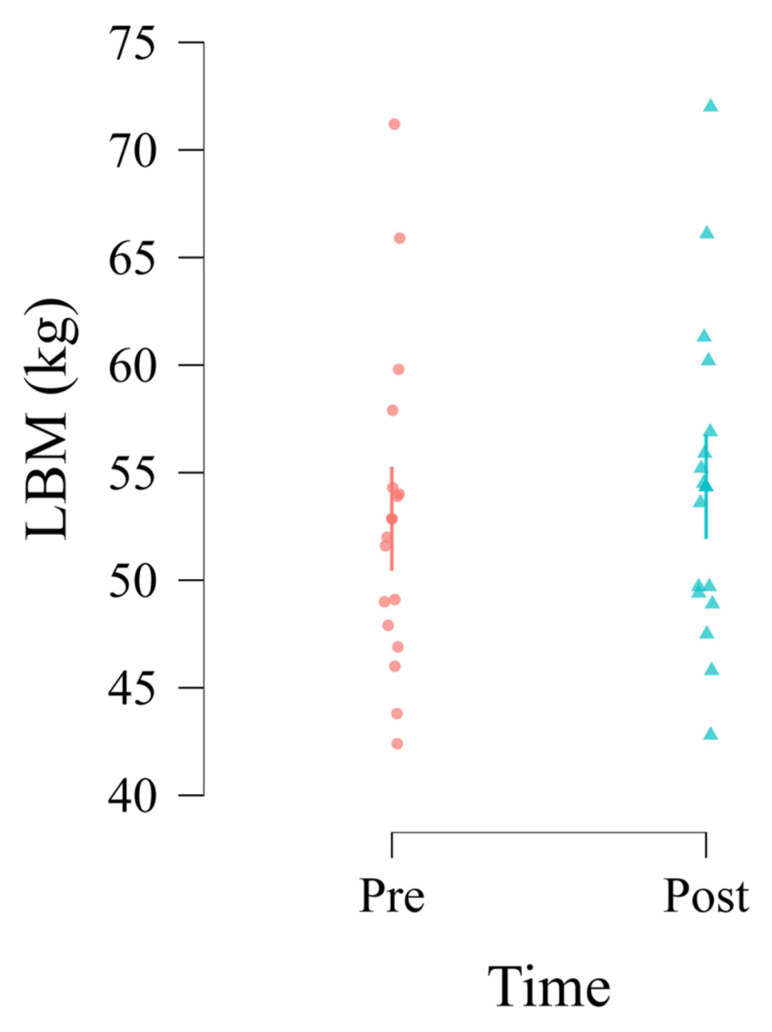
Main effect of Time on lean body mass in kilograms.

The analysis identified a statistically significant main effect of Time on LBM (*F*_1,15_ = 42.56, *p* < 0.001). LBM significantly increased from the Pre-measurement (Estimated Marginal Mean = 52.86 kg, SE = 1.12) to the Post-measurement (Estimated Marginal Mean = 54.34 kg, SE = 1.12). The estimated average increase was 1.49 kg (SE = 0.23, 95% CI [1.02, 1.96], *t*_15_ = 6.52, *p* < 0.001, Cohen’s *d* = 1.63, indicating a very large effect) (Figure 1).

Regarding the covariates, height demonstrated a significant positive association with LBM (Estimate = 1.02, SE = 0.18, *F*_1,13_ = 31.33, *p* < 0.001). This finding suggests that taller individuals tended to exhibit greater LBM, even after controlling for the effects of time and age. Conversely, age did not show a statistically significant relationship with LBM within this model (Estimate = 0.18, SE = 0.66, *F*_1,13_ = 0.08, *p* = 0.785). Collectively, these results indicate that the short-duration pre-season program was associated with a statistically and practically meaningful increase in LBM, independent of age, with taller athletes consistently exhibiting proportionally greater LBM throughout the observation period.

#### 3.2.2. Body Fat Percentage (%)

**Figure 2:**
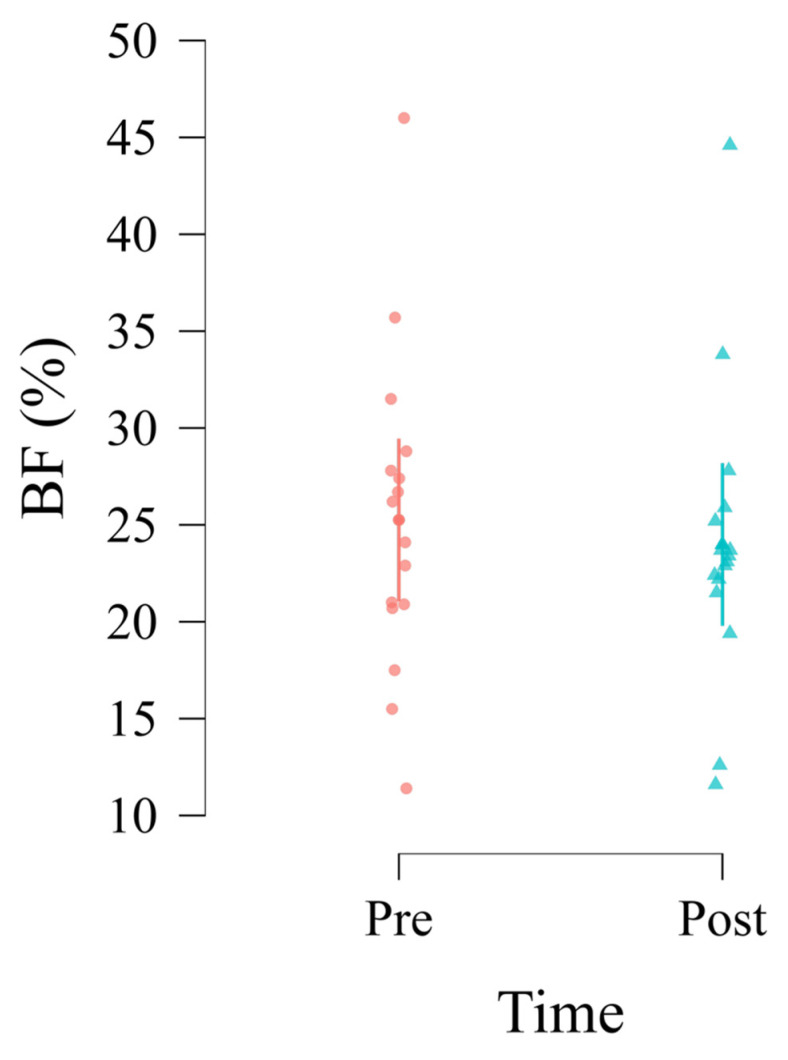
Main effect of Time on body fat percentage.

The LMM for BF% also demonstrated a significant effect of the pre-season program. Model convergence was successfully achieved using the bobyqa optimizer (AIC = 188.64). The conditional R^2^ of 0.96 indicated that the full model, encompassing both fixed and random effects, explained nearly all variance in BF%. Conversely, the marginal R^2^ of 0.14 suggested that the fixed effects accounted for a modest portion of the variance, with between-athlete heterogeneity explaining the substantial remainder. The omnibus test for fixed effects revealed a significant effect of Time (*F*_1,15_ = 4.84, *p* = 0.044), but no significant effects were observed for Age (*F*_1,13_ = 0.22, *p* = 0.649) or Height (*F*_1,13_ = 2.40, *p* = 0.145) (Figure 2).

Parameter estimates showed a significant reduction in BF% from Pre to Post, with an average decrease of 1.27 (SE = 0.58, *t*_15_ = −2.20, *p* = 0.044, 95% CI [−2.45, −0.08], Cohen’s *d* = 0.55, indicating a medium effect). Neither Age (*β* = 0.53, SE = 1.14, *t*_13_ = 0.47, *p* = 0.649, 95% CI [−1.81, 2.88]) nor Height (*β* = 0.49, SE = 0.32, *t*_13_ = 1.55, *p* = 0.145, 95% CI [−0.16, 1.14]) significantly influenced BF%. The random intercept variance (σ^2^ = 58.22, SD = 7.63, ICC = 0.96) indicated considerable individual differences in baseline BF%, with the random effect being statistically significant (LRT = 36.26, *df* = 1, *p* < 0.001).

Post hoc comparisons further confirmed that BF% at Post (Mean = 23.99, SE = 1.95, 95% CI [19.79, 28.18]) was significantly lower than at Pre (Mean = 25.26, SE = 1.95, 95% CI [21.06, 29.45]), with a mean difference of 1.27 (SE = 0.58, *t*_15_ = 2.20, *p* = 0.044). Simple effects analyses indicated that the Time effect was consistent across levels of height and age (*F*_1,15_ = 4.84, *p* = 0.044 for all combinations). No significant Time × Age interaction was observed (*p* = 0.571).

The assumptions underpinning the LMM were adequately met. Residuals exhibited normal distribution (Shapiro–Wilk: *W* = 0.97, *p* = 0.551; Kolmogorov–Smirnov: *D* = 0.12, *p* = 0.732). Visual inspections of Q-Q plots, residual histograms, and residual-predicted scatterplots revealed no significant deviations from normality or homoscedasticity, thereby confirming the suitability of the LMM for these data. Collectively, these findings demonstrate that the short-duration pre-season regimen was associated with a statistically significant, albeit modest, decrease in body fat percentage, independent of age and stature, with residual variance largely attributable to stable individual differences.

## 4. Discussion

This study investigated the impact of a four-week, NCAA Division II compliant pre-season SC program on BC in female collegiate basketball players. The 8 h/week intervention (4 h skill, 4 h SC) yielded statistically significant and favorable changes in both LBM (mean increase: 1.49 kg) and body fat percentage (BF%) (mean decrease: 1.27). While modest in magnitude, the program exerted a very large effect on LBM (Cohen’s *d* = 1.63) and a medium effect on BF% (Cohen’s *d* = 0.55). This disparity suggests a stronger impact on lean mass accretion, indicating considerable clinical relevance. These findings contribute valuable empirical data on time-constrained pre-season program efficacy in NCAA female collegiate basketball players [16,18,24,39]. Results align with our hypothesis that short-term, periodized SC can optimize body composition within stringent NCAA time constraints (8 h/week). This study thus contributes to limited literature on short-duration pre-season programs, underscoring the potential of evidence-based SC strategies to enhance physiological adaptations critical for elite basketball performance.

Contextualizing these results within the existing literature highlights a notable research gap: a paucity of studies specifically evaluating body composition changes across isolated periodization phases (e.g., pre-season, in-season, or post-season) in collegiate women’s basketball. Generally, however, seasonal body composition reports on women’s collegiate basketball indicate a reduction in BF% from pre- to post-season [7,26,71]. Consequently, comparisons herein will be scrutinized from this perspective, with non-collegiate references occasionally presented for broader context [1,7,9,16,17,23,25,27,28,71,72,73].

### 4.1. Changes in Lean Body Mass

The substantial LBM increase (+1.49 kg) observed herein aligns with, and occasionally exceeds, gains reported in other women’s basketball studies, though many span longer durations or different seasonal phases. Our results corroborate Siders et al.’s [27], who reported a 2.0 kg LBM increase by season’s end in players via hydrodensitometry. Similarly, an eight-week supervised exercise program (4 days/week) with whey protein or casein in 16 Division III women’s basketball yielded LBM increases of 1.5 kg and 1.4 kg, respectively, via DEXA [72]. Our findings appear more pronounced compared to a larger study (*n* = 38) where players’ LBM increased by only 0.3 kg (and 0.2 kg for race/ethnicity-adjusted LBM) between pre-season and post-season [26]. Conversely, our results contrast with a larger (*n* = 29) contemporary study on Division I, which reported more varied LBM changes: a 0.9 kg increase from pre-season to in-season, followed by a 1.9 kg decrease from in-season to off-season, assessed by air displacement plethysmography (BOD POD) [24]. Similarly, an international study on elite national athletes, using skinfolds, reported no LBM changes pre-season, mid-season, or post-season, further contrasting our findings [20].

The magnitude of LBM gain in our relatively short intervention suggests a well-structured pre-season program can be highly effective for LBM accretion in female athletes, even under tight scheduling [15,16,23]. Such programs likely incorporate intensified microcycles, potentially featuring Olympic lifting and high-intensity conditioning [6,34,74]. This concentrated gain may signify heightened adaptive potential before the potentially catabolic demands or reduced training focus characteristic of the competitive season.

### 4.2. Changes in Body Fat Percentage

Our results are supported by a substantial systematic review and meta-analysis reporting an average BF% of 20.4 (95% CI [19.4]) for collegiate athletes [18]. The observed significant BF% decrease (−1.27) also resonates with existing literature, albeit with variability. For instance, Kilinc found no significant BF% change after a 10-week program in non-NCAA elite players (skinfolds; control: Δ_change_ (pre-post): 0.6 ± −0.1; basketball: Δ_change_ (pre-post): 1.2 ± 0.3), though study duration and population differed. Our findings align more closely with significant BF% decreases reported across various phases in other studies [75]. Carbuhn et al. noted significant BF% reductions in Division I basketball players from off-season to pre-season and post-season (DEXA) [25]. Similarly, Siders et al. reported a 2.6 kg fat loss over a season [27], and Markos et al. documented a 2.8 BF% decrease from off-season to in-season [73]. Wilborn et al. also demonstrated significant BF% decreases (1–2) with combined training and protein supplementation over eight weeks [72]. Furthermore, our results are consistent with an eight-week pre-season training program for Division II athletes focused on increasing overall strength, which resulted in a BF% decrease from pre (27.25 ± 3.85) to post (24.21 ± 3.12) [29]. Even at the international level, Mexis et al. reported a significant decrease of 1.18 ± 1.32 (skinfolds) during a six-week pre-season [17]. Similarly, when three pre-season training protocols with different frequencies were examined, BF% decreased for the heavy- (−0.3 ± 0.32) and moderate- (−1.29 ± 0.59) frequency training groups, but not light-frequency. Comparable to our study, other authors noted that this BF% reduction was not accompanied by weight loss, suggesting body composition changes occurred through muscle mass accretion [16].

However, some studies report non-significant changes or even BF% increases. Ladwig et al. found a non-significant 1.84 BF% decrease in Division I players, potentially influenced by wider initial BF% range, different training emphases, or measurement variability [28]. Fields et al. reported slight BF% increases across seasonal phases [24]. In stark contrast to the findings of this investigation, a recent international study by Papaevangelou et al. observed no significant BF% alterations in elite-level basketball athletes using skinfolds at pre-season, mid-season, and post-season, presenting a divergent outcome [20].

The modest yet significant BF% reduction (−1.27) within a mere four weeks highlights pre-season conditioning’s considerable potential to impact adiposity. Program intensity likely facilitated this change, though longer interventions or integrated nutritional strategies might be necessary for more substantial fat loss [35,72]. Discrepancy with studies showing no change, as noted by Zouita et al. in a systematic review reporting no body mass/BF% change from resistance/plyometric studies, may underscore the crucial role of specific training program composition and intensity, even over short durations [36].

### 4.3. Model Insights and Covariate Effects

The linear mixed models effectively captured body composition variance, evidenced by high conditional *R*^2^ values (LBM: 0.99; BF%: 0.96). Substantial random intercept variances (ICC: LBM 0.98, BF% 0.96) underscore considerable inter-individual differences in baseline body composition and training responsiveness. This pronounced variability highlights the necessity of individualized training and nutritional approaches, as genetic predispositions, dietary habits, and adherence can significantly modulate outcomes [76,77].

The lower marginal *R*^2^ for BF% (0.14) versus LBM (0.68) suggests fixed effects (Time, Age, Height) explained less BF% variance. This may stem from greater inherent inter-individual variability in BF% regulation or known limitations of BIA in accurately quantifying adiposity, particularly concerning its predictive equations [31,32,78].

The absence of significant ‘Age’ effects on LBM or BF% is unsurprising given the narrow collegiate athlete age range (18–24 years) and short intervention, which may be insufficient for age-related anthropometric differences to manifest [26]. Conversely, ‘Height’ positively associated with LBM, indicating taller individuals possessed greater lean mass, consistent with literature linking anthropometrics to basketball players’ body composition [19,22]. Importantly, ‘Height’ did not significantly influence BF%, aligning with research suggesting BF% in athletic populations is more dynamically influenced by training and dietary factors than stature alone [18,22].

### 4.4. Practical Applications

From a practical standpoint, the observed LBM increase and concurrent BF% reduction are highly relevant for NCAA Division II women’s basketball. Improved BC (greater lean mass, reduced fat mass) can improve power-to-weight ratio, strength, endurance, and potentially injury resilience—all critical for competitive performance [9,20]. These findings offer SC coaches with robust empirical support for implementing intensive, short-duration pre-season programs within NCAA regulatory frameworks. This addresses a notable gap, as few studies isolate effects of such constrained interventions [6]. The results unequivocally suggest coaches can effectively leverage this limited window to induce positive physiological adaptations. By strategically integrating high-intensity resistance training and appropriate conditioning, coaches can transform this seemingly restrictive period into a valuable opportunity to significantly enhance athlete readiness for competitive season demands.

### 4.5. Key Takeways for Coaches

Based on our findings from a 4-week, NCAA-compliant program, we recommend the following evidence-based strategies for S&C coaches:

- Capitalize on the Pre-Season as a High-Impact Window. View the short pre-season not as a constraint, but as a critical opportunity. Our data show that significant, positive changes in body composition (~1.5 kg LBM gain, ~1.3% BF loss) are achievable in just four weeks, setting a strong physiological foundation for the competitive season.
- Prioritize High-Intensity, High-Quality Training. The key to driving these changes is training intensity and quality, not just volume.
  ○ Resistance Training Focus: Center your program on compound lifts (e.g., squats, deadlifts, Olympic lift variations) in the 75–85% 1RM range. Pair these with low-volume plyometrics (e.g., box jumps) to enhance the power-to-weight ratio.
  ○ Session Density: To comply with NCAA time limits (e.g., 4 h/week of S&C), structure sessions around exercise density. Utilize supersets and manage rest periods to keep sessions concise and effective (e.g., 3–4 sessions of ‚â§45–60 min each).
- Link Training to On-Court Performance. Clearly communicate the “why” behind the training. Emphasize that favorable changes in body composition directly support on-court strength, endurance, and potential injury resilience.
  ○ Pragmatic Monitoring: While DEXA is the gold standard for annual validation, use practical tools like BIA to track body composition trends throughout the pre-season.
  ○ Performance Proxies: Supplement body composition data with performance metrics. Improvements in vertical jump, sprint times, or agility tests can serve as excellent proxies for an enhanced power-to-weight ratio.

### 4.6. Limitations and Future Directions

This study possesses several limitations inherent to its applied, observational design. The sample size (*n* = 16), while common in sport science and adequate to detect the reported effects, was a convenience sample dictated by NCAA regulations. This, along with the pronounced interindividual variability observed (ICC > 0.96), limits the generalizability of the findings. Furthermore, the absence of a non-training control group, though ethically and logistically challenging in elite athletic settings, prevents definitive causal attribution of the observed changes solely to the intervention. While the sensitivity power analysis indicated sufficient power for large effects and LMMs are robust for smaller samples, future multi-institutional studies employing controlled designs are necessary to strengthen causal inferences and enhance replicability [79].

Reliance on BIA for body composition assessment, while practical for field settings, is a limitation. BIA is susceptible to hydration status and may provide different absolute values compared to gold-standard methods like DEXA, potentially underestimating BF% and overestimating LBM in some populations [18,31,80]. Future research should aim to validate such findings using DEXA to improve measurement precision.

Furthermore, the observational design precluded full control over external variables that influence body composition, such as dietary intake (e.g., protein, caffeine), low energy availability, sleep patterns, and psychological status [76,81,82,83,84,85]. In particular, dietary intake was not standardized, and the potential influence of the menstrual cycle phase, a significant variable affecting fluid balance, metabolism, and hormonal status in female athletes, was not accounted for [36,86,87,88,89]. Future research should integrate detailed dietary analysis and track menstrual cycle phase to isolate the training effect more precisely.

Specific SC program details could not be fully disclosed, limiting direct replicability, though demonstrated effectiveness points to sound underlying principles [18,34,74,79,90]. The lower marginal *R*^2^ for BF% suggests other unmeasured variables (e.g., diet, genetics) significantly contributed to its variance, warranting their inclusion in future, more rigorously controlled experimental designs [9,71].

Future investigations should consider longitudinal tracking to determine if pre-season body composition changes persist, augment, or attenuate throughout the competitive season and into the off-season [7,16,26,71]. Critically, such studies should integrate direct measures of athletic performance (e.g., vertical jump, sprint times, change-of-direction speed) to establish a functional link between body composition changes and on-court capabilities [21]. Integrating detailed dietary analysis, hormonal profiling (e.g., estradiol, testosterone), and direct measures of athletic performance would provide a more comprehensive understanding of mechanisms driving these changes and their functional consequences [5,7,16,21,26,42,43,44,71,85,89,91].

## 5. Conclusions

In conclusion, this study unequivocally demonstrates that a four-week, NCAA-compliant pre-season strength and conditioning program can induce statistically significant and practically meaningful improvements in body composition, specifically, a very large increase in LBM and a medium decrease in BF%, among NCAA Division II female basketball players. These findings underscore the capacity for well-designed, intensive training protocols to optimize physiological preparedness even within restrictive timeframes. While these promising results should be interpreted with caution due to the observational design and lack of a control group, the results hold considerable practical value for strength and conditioning professionals aiming to maximize athlete development during the crucial pre-season window. By strategically integrating high-intensity resistance training and appropriate conditioning, coaches can transform this seemingly restrictive period not into a constraint, but into a valuable opportunity to significantly enhance athlete readiness for the rigorous demands of the competitive season.

## Figures and Tables

**Table 1:** Descriptive and variability metrics of study variables.

			95% Confidence Interval Mean			95% Confidence Interval Std. Dev.	
	Mean	Std. Error of Mean	Upper	Lower	Std. Deviation	Upper	Lower
Age (y)	20.56	0.45	21.52	19.61	1.79	2.77	1.32
Height (cm)	173.88	1.61	177.32	170.43	6.46	10.00	4.77
Body mass (kg)							
Pre	76.19	5.04	86.93	65.45	20.16	31.20	14.89
Post	76.83	4.84	87.14	66.51	19.36	29.97	14.30
BF%							
Pre	25.26	2.06	29.64	20.87	8.23	12.74	6.08
Post	23.99	1.89	28.02	19.95	7.57	11.71	5.59
LBM (kg)							
Pre	52.86	1.95	57.02	48.70	7.81	12.08	5.77
Post	54.34	1.93	58.46	50.23	7.72	11.94	5.70

Note: BF%: body fat percentage; LBM: lean body mass.

## Data Availability

The raw data supporting the conclusions of this article will be made available by the authors on request.

## Abbreviations

The following abbreviations are used in this manuscript:

NCAA	National Collegiate Athletic Association
SC	Strength and conditioning
BC	Body composition
LD	Linear dichroism
LBM	Lean body mass
BF%	Body fat percentage
DEXA	Dual energy X-ray absorptiometry
BIA	Bioelectrical impedance analysis
LMM	Linear mixed model
REML	Restricted maximum likelihood
BODPOD	Air displacement plethysmography
